# Predicting Molecular Energies of Small Organic Molecules With Multi‐Fidelity Methods

**DOI:** 10.1002/jcc.70056

**Published:** 2025-03-04

**Authors:** Vivin Vinod, Dongyu Lyu, Marcel Ruth, Peter R. Schreiner, Ulrich Kleinekathöfer, Peter Zaspel

**Affiliations:** ^1^ School of Mathematics and Natural Sciences University of Wuppertal Wuppertal Germany; ^2^ School of Science Constructor University Bremen Germany; ^3^ Institute of Organic Chemistry Justus Liebig University Giessen Germany

**Keywords:** atmopsheric chemistry, cost efficiency, coupled cluster, delta learning, density functional theory, machine learning, multi‐fidelity data, organic chemistry, quantum chemistry, sparse data

## Abstract

Multi‐fidelity methods in machine learning (ML) have seen increasing usage for the prediction of quantum chemical properties. These methods, such as Δ‐ML and Multifidelity Machine Learning (MFML), have been shown to significantly reduce the computational cost of generating training data. This work implements and analyzes several multi‐fidelity methods including Δ‐ML and MFML for the prediction of electronic molecular energies at DLPNO‐CCSD(T) level, that is, at the level of coupled cluster theory including single and double excitations and perturbative triples corrections. The models for small organic molecules are evaluated not only on the basis of accuracy of prediction, but also on efficiency in terms of the time‐cost of generating training data. In addition, the models are evaluated for the prediction of energies for molecules sampled from a public dataset, in particular for atmospherically relevant molecules, isomeric compounds, and highly conjugated complex molecules.

## Graphical abstract

Multi‐fidelity methods in machine learning have seen increasing usage for the prediction of quantum chemical properties. This work implements and analyzes several multi‐fidelity methods for the prediction of electronic molecular energies at the level of coupled cluster theory including single and double excitations and perturbative triples corrections. The models are studied based on the cost of generating training data and the accuracy of prediction for atmospherically relevant molecules, isomeric compounds, and highly conjugated complex molecules.

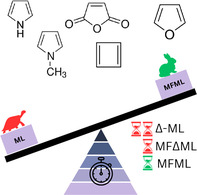

## 1 Introduction

High‐accuracy quantum chemistry (QC) computations are integral to understanding day‐to‐day processes. One of these is, for example, the use of high‐accuracy thermochemical calculations to understand atmospheric chemistry. Coupled cluster theory with single, double, and a perturbative treatment of triple excitations (CCSD(T)) is widely regarded as the “gold standard” in quantum chemistry for accurately describing electron correlation in molecular systems [1]. By incorporating perturbative triple excitations on top of the CCSD wave function, CCSD(T) achieves a higher level of accuracy in predicting molecular properties such as reaction energies and potential energy surfaces. However, this accuracy also comes at a high computational cost, as the CCSD(T) method scales approximately as $O^{2}(N^{8})$ with the number of basis functions N and occupied orbitals O, which makes it impossible to be applied to larger systems. Several approximations have been employed to overcome this scaling problem [2], and one efficient approach is the domain‐based local pair natural orbital (DLPNO) approximation [3]. This method reduces the computational cost by localizing electron correlation to spatially compact regions of the molecule, without significantly compromising accuracy. Although it reduces the computational cost by a factor of two to four compared to ordinary coupled cluster calculations [4, 5], it is still computationally challenging for large systems.

The use of ML in QC has significantly reduced the computational cost for large chemical systems [6, 7, 8, 9, 10, 11, 12, 13]. The ML models learn a mapping between the Cartesian coordinates along with the respective atomic number, often converted to machine learnable input features called *molecular descriptors* or *representations*, and the QC property of interest such as ground state energies. This allows them to make predictions of the QC properties for molecules that the model has not been trained on. While ML in QC has provided a major respite to the cost of making costly calculations, a new overhead has since been presented to the use‐case of ML‐QC pipelines. This is the cost of generating the training data required for an ML model to achieve a certain accuracy. It is a common observation that the more training samples one uses, the better the model is able to predict the QC property of interest [11, 14].

One method to reduce the cost of training data is the Δ‐ML method [15]. In Δ‐ML, training data from two different fidelities are used to train an ML model on the difference between the two fidelities. It is observed with the application of Δ‐ML based methods that it is easier to learn the difference rather than the explicit value at the highest fidelity [11, 12, 14, 15]. The final prediction with an Δ‐ML model involves the QC calculation of the cheap fidelity and the prediction of the difference. Since its introduction in the QC community, it has become a ubiquitous tool for a vast array of applications, including excitation energies, potential energy surfaces, electronic spectra, and isomerization enthalpies [11, 12, 14, 15, 16, 17, 18, 19, 20]. The method demonstrated that a smaller number of training samples could be used to achieve a higher level of accuracy in the model. Previously, in [20] some of the present authors used the Δ‐ML approach to learn the CCSD(T) corrections over the CCSD energies for a collection of small organic molecules. In another related work, the Δ‐ML was employed to predict the CCSD(T) energies of small organic monomers based on DFT results [21]. It is to be noted that Δ‐ML is slightly different from transfer learning (TL) [22] which is another common approach used in ML‐QC to reduce the use of costly data and has been employed in diverse applications such as thermochemistry and material analysis [23, 24, 25]. The key difference is that while Δ‐ML trains on the explicit difference between two fidelities, TL first trains an ML model on the low fidelity and uses that to train for model parameters such as in the case of a neural network, the weights of the different hidden layers. The model parameters from this cheap‐fidelity network are then “transferred” to a new model, which is trained on the sparsely available high‐fidelity data.

A systematic generalization of the Δ‐ML method towards the use of data from multiple fidelities in machine learning, named CQML, was introduced in [26]. In this method, an ML model is trained on several fidelities that lie between the top fidelity, also termed *target fidelity*, and the cheaper fidelity. In addition, this approach eliminates the need to perform QC calculations at the cheapest fidelity, also called the *baseline fidelity*. CQML is, hence, a method for multi‐fidelity machine learning (MFML). MFML methods have been used in several applications such as the prediction of atomization energies at the CCSD(T) level for a diverse range of molecules [26], predicting bandgaps [27, 28], and excitation energies along molecular trajectories [29] among others [30, 31]. In the following, we will refer to the multilevel method discussed in [29] as the *MFML* method. Alternative variations of the Δ‐ML and MFML method have been introduced. Hierarchical‐ML (hML) builds several Δ‐ML‐like models for different fidelities in a manner similar to an MFML approach, however, with the number of training samples chosen to use an *ad hoc* optimization scheme [32]. The method has been shown to be effective in predicting ground state potential energy surfaces for $\text{C}{\text{H}}_{3}\text{C}\text{l}$. Optimized MFML (o‐MFML), was recently introduced as a methodological improvement over the conventional MFML approach by optimally combining the sub‐models used for MFML [33]. The o‐MFML method uses a validation set computed at the target fidelity to optimize the combination of the sub‐models and has been shown to provide better accuracy for the overall prediction for both excitation energies and atomization energies [33] and in cases where training data might be heterogeneous [34]. Multi‐task Gaussian processes are yet another method introduced recently and have been seen to reduce the overall cost associated with a multi‐fidelity model [35]. The model was seen to be effective in the prediction of many‐body interaction terms for water and showed favorable results even in cases of heterogeneous training data. Another useful approach to reduce the cost of training data is the recently introduced minimal multilevel machine learning (M3L) method, an update of the MFML method. In this method, the number of training samples to be used at each fidelity is optimally computed using Bayesian optimization of a cost function for a target model error [36].

A recent study benchmarked different multi‐fidelity models with respect to the time‐cost associated with them and the corresponding model accuracy [37]. This study established that the use of MFML is beneficial when requiring large numbers of predictions. It also introduced a new multi‐fidelity approach, the multi‐fidelity Δ‐ML (MFΔML) method. In this method, several Δ‐ML‐like sub‐models are combined in a manner similar to that in MFML. This method was shown to be superior to the conventional Δ‐ML method in model error and overall efficiency. Vinod and Zaspel [37] performed these benchmarks for models that are trained and evaluated across different fidelities restricted to the DFT level of theory.

One possible application for high‐accuracy thermochemistry is the domain of atmospheric chemistry, also including large‐scale climate models that consider chemical processes [38]. Atmospheric chemistry encompasses a multitude of gas‐phase radical reactions, most of which are not amenable to experiment. Therefore, a precise prediction of their relative energies is paramount. In this study, a database of small organic molecules containing multiple free radicals, and their hydrogen‐terminated counterparts were constructed. Several multi‐fidelity methods were used to train ML models and evaluate this collection of monomers. Subsequently, these models were evaluated not only on their accuracy of predictions but also on the cost associated with training them, in particular, the cost of the training data required to achieve a certain error. Finally, all models were assessed on supplementary validation datasets, comprising manually selected atmospherically pertinent molecules, highly conjugated molecules, and isomer structures. The latter category represents a particularly challenging theoretical distinction.

The rest of the manuscript is structured as follows: The required methodological details are provided in Section 2 including QC methods and ML techniques. Section 3 assesses the different ML methods discussed in this work for the prediction of the molecular energies at the DLPNO‐CCSD(T) target fidelity. A time‐cost versus model accuracy assessment is presented to gauge the effectiveness of each of the studied ML methods. Conclusions are drawn from the results, and special cases are studied in detail and discussed in Section 4. An outlook and key takeaway messages of this work are delineated in Section 5.

## 2 Method

### 2.1 Dataset Construction

We extended the database from a previous study [21], where around 8000 monomers were randomly selected from a public database that focuses on determining the enthalpies of radical reactions for small organic molecules [39]. These were then geometry optimized at the B3LYP‐D3(BJ)/cc‐pVTZ level of theory and their single‐point energies were computed using DLPNO‐CCSD(T) theory. More than 12,000 additional molecules from the same QC database were geometry optimized at the B3LYP‐D3(BJ)/cc‐pVTZ level of theory. The free radicals in the database are important intermediates in combustion and atmospheric chemistry and their energies are essential to determine the thermodynamics and kinetics of reaction pathways. In order to save time and cost for advanced quantum chemical calculations, we only selected small molecules in the database (no more than ten heavy atoms). The molecular energy and weight distributions of our dataset are given in the supplementary information. After checking for duplicates via the generated SMILES, 12,340 molecules remained in our database (4606 data points with DLPNO‐CCSD(T) single‐point energies from the previous database and 7734 additional molecules) consisting of only hydrogen, carbon, nitrogen, and oxygen atoms. All these molecules were then subjected to DFT single‐point energy computations using the B3LYP‐D3(BJ) functional in conjunction with the STO‐3G basis set. Subsequently, 1500 data points with DLPNO‐CCSD(T) energies were randomly selected as the test set for our ML models, and all the rest were used for training. In addition, we validated our models using three external validation sets containing atmospherically relevant species (including radicals), highly conjugated molecules, and isomers, which were also used for validation in the previous study [21].

### 2.2 Machine Learning Methods

This subsection discusses the ML approaches used in this work, including the single‐fidelity model, the Δ‐ML approach, and MFML with its variants.

#### 2.2.1 Molecular Descriptors

In the general ML‐QC pipeline, an integral part of the process of learning a QC property is to first convert the Cartesian coordinates and atomic numbers of atoms of the molecules into machine learnable input features, which are called *representation* or *molecular descriptors* [11, 14, 40]. A variety of such descriptors exist in the literature with each suited for a specific application. A molecular descriptor is expected to satisfy certain conditions such as uniqueness, rotational, and translation invariance, and invariance under different indexing of the atoms. Rotation and translation invariance can be understood as follows: If a molecule is moved or rotated in a global coordinate system, its energy does not change. A good molecular descriptor should be able to reflect this.

The use of unsorted Coulomb Matrices (CM) [6, 41] with geometries of different molecules while being translation and rotation invariant lacks the index invariance [42]. This issue can be mitigated by the use of row‐sorted CM wherein the unsorted CM is built, then the rows are ordered by their $L_{2}$ norms [6, 40]. However, sorting of CM is generally considered to introduce undesirable discontinuities [6, 41]. In order to combat the issues of index invariance, several variants of CM have been suggested. Other alternatives that exist include using a different distance metric while building the kernel function with unsorted CM, namely the Wasserstein distance, which is the lowest amount of work done to change one distribution to another [43]. Even with unsorted CM, this metric has lower ML model errors than the $L_{2}$ and $L_{1}$ distance metrics. Yet another proposition to overcome the index invariance issues of unsorted CM is the use of Randomized CMs as shown to be effective in the prediction of molecular electronic properties [7]. Other molecular descriptors such as Spectral London and Axilrod‐Teller‐Muto (SLATM) [44, 45], smooth overlap of atomic potential (SOAP) descriptors [46], and the Faber‐Christensen‐Huang‐Lilienfeld representation (FCHL) [47] satisfy the index invariance in addition to the other requirements of a molecular descriptor. These descriptors build more chemistry‐informed descriptors and have been employed in several used cases and shown to be effective.

Since the aim of this work is not to provide a thorough review of the descriptors, three common representations were initially studied, namely: CM, row‐sorted CM, and the SLATM. All three descriptors were generated using the qmlcode package [48]. The parameters for the SLATM representation used in this work were set to the values prescribed in [44], namely: A cut‐off radius of 4.8 Å, a smear width of 0.05 Å for the radial terms and 0.05 rad as value for the angular terms. In this work, the default London potential was employed in the generation of the SLATM representation. These values were chosen in order to prevent overfitting of the ML models to the training dataset and make the transfer of the models to the additional validation sets as feasible as possible. The values employed in [44] indicate that these can be applied globally for most molecules, as is the case for this present work.

#### 2.2.2 Kernel Ridge Regression

The predictions of a kernel ridge regression (KRR) model for a given fidelity f is given as

(1) $$P_{\text{KRR}}^{\left(f\right)}\left(X_{q}\right):\;=\overset{N_{\text{train}}^{\left(f\right)}}{\underset{i=1}{\sum }}\alpha_{i}^{\left(f\right)}k\left(X_{q},X_{i}\right)$$

where k(·,·) denotes the kernel function and $N_{\text{train}}^{\left(f\right)}$ the number of training samples used at the fidelity f. This work uses the Matérn kernel of second‐order with $l_{2}$ norm which is computed as

(2) $$\begin{array}{c} k\left(X_{i},X_{j}\right)=\exp\left(-\frac{\sqrt{3}\cdot d_{\text{ij}}}{\sigma }\right)\cdot \left(1+\frac{\sqrt{3}\cdot d_{\text{ij}}}{\sigma }\right) \end{array}$$

where the parameter σ is a length scale and $d_{\text{ij}}\equiv {\left\|X_{i}-X_{j}\right\|}_{2}^{2}$. In this work, using a grid search, the parameter σ was optimized to values of 3200.0 for SLATM, 9000.0 for sorted CM, and 9500.0 for unsorted CM. The hyper‐parameter grid search for σ was carried out only for the target fidelity of DLPNO‐CCSD(T). The vector $\alpha^{\left(f\right)}$ contains the coefficients of KRR, which are calculated by solving the linear system $(K+\lambda I)\alpha^{\left(f\right)}=y^{\left(f\right)}$. Here, $K={\left(k(X_{i},X_{j})\right)}_{i,j=1}^{N_{\text{train}}}$ is referred to as the kernel matrix. The vector $y^{\left(f\right)}={\left(y_{1}^{\left(f\right)},y_{2}^{\left(f\right)},\text{…},y_{N_{\text{train}}}^{\left(f\right)}\right)}^{T}$ is the vector of QC properties, in the present case the energies, from the training set denoted as $T^{\left(f\right)}$. The parameter λ restricts the overfitting of the model and was set in this work to $10^{-10}$.

#### 2.2.3 Δ‐Machine Learning

Let $T^{F}:\;={\left\{(X_{i},y_{i}^{F})\right\}}_{i=1}^{N_{\text{train}}^{F}}$ be training data computed at some fidelity F which is supposed to be the final prediction fidelity, that is, the target fidelity. Here $X_{i}$ are molecular descriptors with $y_{i}$ corresponding QC‐properties. For the same molecular descriptors, let a training set of QC calculations made at a cheaper fidelity $f_{b}^{QC}<F$ be given: $T^{f_{b}^{QC}}:\;={\left\{(X_{i},y_{i}^{f_{b}^{QC}})\right\}}_{i=1}^{N_{\text{train}}^{F}}$. Notice that the training set $T^{f_{b}^{QC}}$ has the same number of samples as the set $T^{F}$, by construction. With these training datasets at two fidelities, the prediction of a Δ‐ML model for the target fidelity is given as

(3) $$P_{\Delta }^{\left(F;f_{b}^{QC}\right)}:\;=P_{\text{KRR}}^{\Delta_{f_{b}^{QC}}^{F}}(X_{q})+y_{q}^{f_{b}^{QC}}$$

where, $P_{\text{KRR}}^{\Delta_{f_{b}^{QC}}^{F}}$ denotes the KRR prediction of the energy difference between the two fidelities, and $y_{q}^{f_{b}^{QC}}$ is the QC‐calculation for the query molecule.

#### 2.2.4 Multi‐Fidelity Machine Learning

The MFML approach was introduced as a systematic generalization of the Δ‐ML method [26]. The method iteratively uses *sub‐models* of KRR. The sub‐models are identified by the fidelity, f, and the number of training samples used in that fidelity, $2^{\eta_{f}}=N_{\text{train}}^{\left(f\right)}$ for KRR. That is, sub‐models can be identified by a composite index $s=(f,\eta_{f})$. The sub‐models for a given MFML model are chosen based on the choice of the number of training samples at the target fidelity and the *baseline fidelity*, which is the cheapest QC fidelity that is included in the MFML model [26, 33]. The prediction from an MFML model is given as

(4) $$P_{\text{MFML}}^{\left(F,\eta_{F};f_{b}\right)}\left(X_{q}\right):\;=\underset{s\in S^{\left(F,\eta_{F};f_{b}\right)}}{\sum }\beta_{s}P_{\text{KRR}}^{\left(s\right)}\left(X_{q}\right)$$

The summation runs over the set of MFML sub‐models, $S^{\left(F,\eta_{F};f_{b}\right)}$. Notice that the prediction of an MFML model does not require any further QC calculations to be performed during evaluation, unlike in the case of Δ‐ML as seen in Equation (3). The $\beta_{s}$ from Equation (4) are coefficients of MFML that are set to

(5) $$\beta_{s}^{\text{MFML}}=\left\{\begin{array}{ll} +1,\; & \text{if}\;f+\eta_{f}=F+\eta_{F}\\ -1, & \text{otherwise} \end{array}\right.$$

An alternative formulation of the coefficients is introduced in [33]. This results in the optimized multi‐fidelity machine learning approach (o‐MFML). This method computes optimal values of $\beta_{s}$ by solving the following optimization problem

$$\begin{array}{c} \beta_{s}^{\text{opt}}=\arg\underset{\beta_{s}}{\min}{\left\|\overset{N_{\text{val}}}{\underset{v=1}{\sum }}\left(y_{v}^{\text{val}}-\underset{s\in S^{\left(F,\eta_{F};f_{b}\right)}}{\sum }\beta_{s}P_{\text{KRR}}^{\left(s\right)}\left(X_{v}^{\text{val}}\right)\right)\right\|}_{p} \end{array}$$

This procedure is carried out over a validation set given as $V_{\text{val}}^{F}:={\left\{(X_{q}^{\text{val}},y_{q}^{\text{val}})\right\}}_{q=1}^{N_{\text{val}}}$. The validation set consists of geometries with the energies computed at the target fidelity. That is, the training of the o‐MFML model comes with the additional cost of the validation set. The prediction of the o‐MFML model for query descriptor $X_{q}$ is given as

(6) $$\begin{array}{c} P_{o-\mathrm{MFML}}^{\left(F,\eta_{F};f_{b}\right)}\left(X_{q}\right):\;=\underset{s\in S^{\left(F,\eta_{F};f_{b}\right)}}{\sum }\beta_{s}^{\text{opt}}P_{\text{KRR}}^{\left(s\right)}\left(X_{q}\right) \end{array}$$

where $\beta_{s}^{\text{opt}}$ are the optimized coefficients.

#### 2.2.5 Multi‐Fidelity Δ‐Machine Learning

Consider an ordered hierarchy of fidelities, f∈{1,2,…,F}, such as that used for MFML. With such a hierarchy, all the training energies can be “centered” by the energies of the lowest fidelity, f=1. These can then be used to build a MFML model. That is, the sub‐models are now individual Δ‐ML models. This formulation is referred to as the multi‐fidelity Δ machine learning (MFΔML) approach [37]. For a query representation $X_{q}$, the prediction of the MFΔML model is given as:

(7) $$P_{MF\Delta ML}^{\left(F,\eta_{F};f_{b},f_{b}^{QC}\right)}(X_{q}):\;=\underset{s\in S^{\left(F,\eta_{F};f_{b}\right)}}{\sum }\beta_{s}P_{\Delta }^{\left(s\right)}\left(X_{q}\right)$$

where, $P_{\Delta }^{\left(s\right)}$ are Δ‐ML models from Equation (3) where $f_{b}^{QC}$ is set to f=1, and the target fidelity for each sub‐model would be fidelity f. Note that each evaluation of this model requires a QC calculation on the lowest level.

#### 2.2.6 Model Error Metrics

In order to determine the accuracy of the ML models studied in this work, the mean absolute error (MAE) of the predictions over a holdout test set was studied. Consider the test set of query molecular descriptors and corresponding energies computed at the target fidelity, $T_{\text{test}}:\;={\left\{(X_{q},y_{q}^{\text{test}})\right\}}_{q=1}^{N_{\text{test}}}$. The MAE of ML predictions over this test set is computed as

(8) $$\text{MAE}:=\frac{1}{N_{\text{test}}}\overset{N_{\text{test}}}{\underset{q=1}{\sum }}|y_{q}^{\text{test}}-y_{q}^{ML}|$$

where $y^{ML}$ can be the predictions from any of the ML models discussed above. The MAEs of the different ML models are discussed in the form of learning curves, which plot the MAE values as a function of the number of training samples used at the target fidelity for a given ML model. In addition, the MAE of the different models is studied as a function of the time‐cost incurred in generating the complete training data for the model. Consider the case of single‐fidelity KRR. The cost of training data is explicitly related to the number of training samples used at the target fidelity. For the case of the multi‐fidelity methods, this cost will include not just the cost of the target fidelity training samples, but also the cost of the training data used at the subsequent cheap fidelities. For o‐MFML, the cost also includes the expense of the validation dataset at the target fidelity. For the Δ‐ML variants, the cost of the ML model also includes the cost of making the QC‐baseline calculations. The resulting analysis for this is provided in Section 3.

Fully trained MFML and MFΔML models with $N_{\text{train}}^{\mathrm{CCSD}(T)}=512$ are also evaluated on additional validation sets of atmospheric molecules (Atmos), conjugated compounds (Conjugated), and isomeric compounds (Isomers). In these cases, the model error is reported as a single MAE value, and the distribution of the difference between reference and predicted energies is studied with kernel density plots (see Section 4). A simple scatter of the reference and predicted energies is also provided for the sake of completeness.

## 3 Results

**FIGURE 1:**
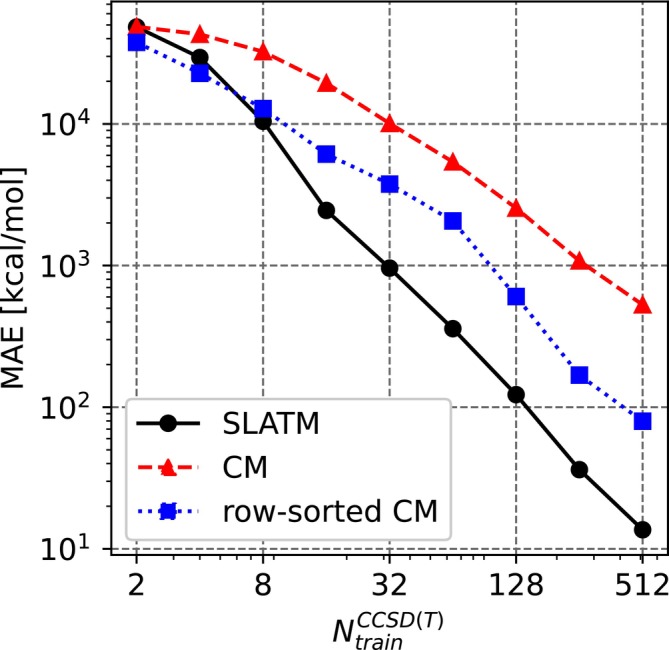
Comparing representations for single‐fidelity KRR at the DLPNO‐CCSD(T) fidelity. Results are shown for an average of ten runs with shuffled training data. The SLATM representation performs the best out of the three, and the sorted Coulomb Matrices (CM) performs better than the unsorted CM.

A preliminary assessment of molecular descriptors was made to prepare for the use of multi‐fidelity methods in the dataset. Unsorted CM, row‐norm sorted CM, and SLATM molecular descriptors were tested since these are the most common descriptors for such applications. The results of the assessment are shown in Figure 1 for a single‐fidelity KRR model trained only on the target fidelity DLPNO‐CCSD(T). The learning curves indicate that the SLATM representation performs the best out of the three. The sorted CM performs better than the unsorted CM. This could be due to the fact that the sorted CM and SLATM representations retain the index invariance of the descriptor, which is missing in the unsorted CM descriptor. For a use case such as the one presented here where the models are trained and evaluated on different molecules as opposed to training on a trajectory of the same molecule as in [29], the retention of indexing invariance is pertinent [6, 11, 40, 49]. At the same time, the sorted CM performs worse than the SLATM representation. This could be due to the fact that the sorting of the CM results in undesirable discontinuities [6, 40] which potentially deter the ML models from being able to learn anything meaningful. Based on this assessment, for the remainder of this work, the SLATM representation is used throughout all ML models. The preliminary data assessment of the training data as prescribed in [29] is given in the supplementary information associated with this manuscript in Figure S2. The analysis indicates that the chosen hierarchy of the fidelities is indeed conducive to the effective working of the multi‐fidelity models. The mean absolute difference in the energy values of the fidelities shows a systematic decrease and is a first indicator of the abilities of the MFML model in predicting the target fidelity with good accuracy.

**FIGURE 2:**
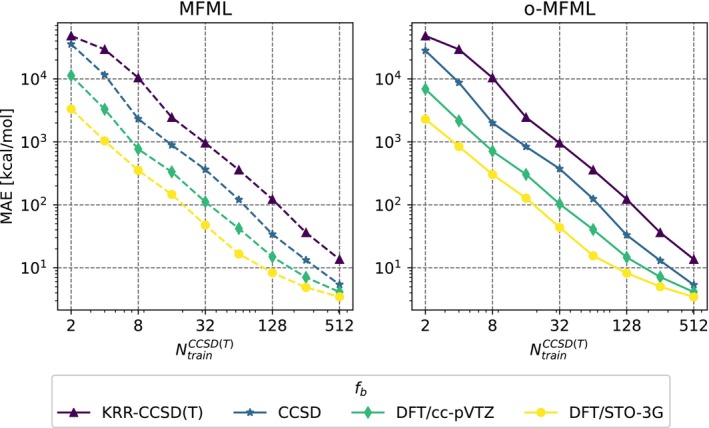
MFML and o‐MFML learning curves with varying baseline fidelities. The learning curve for the single‐fidelity KRR model built with only DLPNO‐CCSD(T) training data is also shown for reference.

MFML and o‐MFML models were built with varying baseline fidelities for the prediction of energies for the monomers. The resulting learning curves are presented in Figure 2 for both these models. The single‐fidelity KRR built with only DLPNO‐CCSD(T) training samples is shown for reference. With the addition of cheaper fidelities, the learning curves of the models show a constant lowered offset. That is, for the same number of training samples as used for the single‐fidelity KRR model, the MFML models result in a lower MAE. While the o‐MFML model is a methodological improvement over the MFML method, in this case, the difference is not very pronounced and the model MAEs for MFML and o‐MFML are rather similar.

**FIGURE 3:**
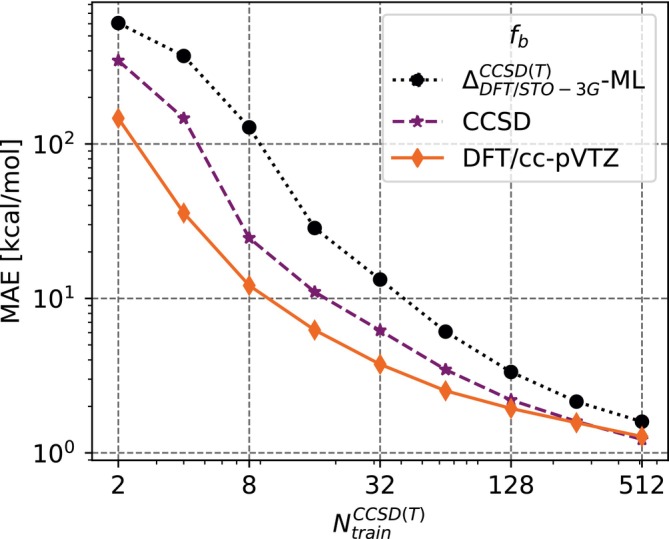
Learning curves for MFΔML. The QC baseline is DFT/STO‐3G. The different baseline fidelities of the MFΔML model are shown in the legend. The learning curve of Δ‐ML model built with DFT/STO‐3G as QC‐baseline and DLPNO‐CCSD(T) target fidelity is also plotted.

In this work, we also assess the Δ‐ML and MFΔML methods. The reader is referred to Figures S3 and S4 in the supplementary information for results of Δ‐ML with different values of $QC_{b}$. The overall trend is as expected based on the study from [15, 37]. That is, with a $QC_{b}$ that is closer to the target fidelity, the Δ‐ML model shows a higher accuracy in prediction. However, as Figure S4 indicates, the time‐cost incurred in using higher $QC_{b}$ far outweighs this benefit. As described in Section 2.2.5, the MFΔML method builds a multi‐fidelity model consisting of various Δ‐ML models. The resulting learning curves are shown in Figure 3. In addition to the learning curves for MFΔML, the learning curve for the standard Δ‐ML model built with the DFT/STO‐3G as QC‐baseline is shown as well. Once again, as for the case of MFML, the addition of a cheaper fidelity to the basic Δ‐ML model results in a lower offset of the learning curve. However, for large enough training set sizes, $N_{\text{train}}^{\mathrm{CCSD}(T)}=512$, this offset is not very pronounced vis‐á‐vis the Δ‐ML model. Furthermore, the learning curve for MFΔML with $f_{b}$ CCSD and $f_{b}$ DFT/cc‐PVTZ converge at this point. This convergence could be an indication of the saturation of the model due to the very similar structures of the monomers.

**FIGURE 4:**
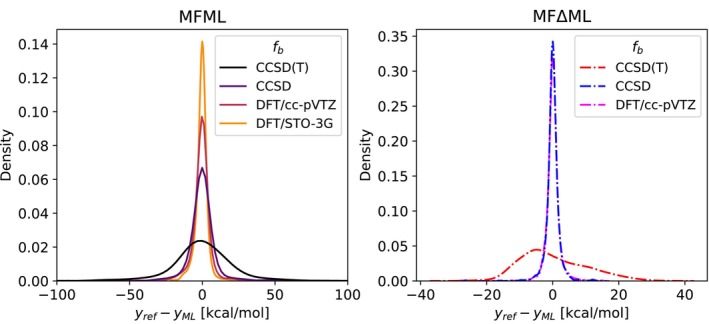
Distribution of difference in model prediction and computed reference DLPNO‐CCSD(T) energies over the holdout test set of 1,500 samples for MFML and MFΔML models with varying values of $f_{b}$.

Figure 4 depicts the difference between ML model prediction and reference DLPNO‐CCSD(T) energies for the holdout test set used for the study of learning curves. The results are shown for both the MFML and MFΔML models with varying baseline fidelities. The error distribution of the single‐fidelity KRR with only DLPNO‐CCSD(T) energies and the standard Δ‐ML model with DFT/STO‐3G as the QC‐baseline are also shown for reference. Consider the left‐hand side plot of Figure 4 which is the case for the single‐fidelity KRR and MFML models. It is seen that all the ML models predict a difference centered around 0 kcal/mol. However, the single‐fidelity KRR model has a wide spread of the difference between reference and prediction. With each additional cheaper fidelity that is added to create the MFML model, the peak of the differences gets tighter around 0 kcal/mol meaning, the MFML models predict the DLPNO‐CCSD(T) energies with increasing accuracy as one decreases the baseline fidelity. This agrees with the study of learning curves that was presented in Figure 2.

The right‐hand side plot of Figure 4 depicts the distribution of the difference between reference DLPNO‐CCSD(T) energies and the energies predicted by the different Δ‐ML models that were studied in this work. These are built with the DFT/STO‐3G fidelity as the QC baseline as explained in Section 2.2.5. Note that the *x*‐axis, marking the differences, is different from that for the MFML models on the left‐hand side plot, almost by an order of magnitude. On comparing the distribution of differences for the different Δ‐ML models, the standard Δ‐ML model (denoted in the legend by the DLPNO‐CCSD(T)) has the widest distribution range with a peak that is shifted towards the left of 0 kcal/mol. With the addition of cheaper baselines to create the MFΔML models, the peak becomes narrower and centered around 0 kcal/mol. This is once again in agreement with the analysis of the learning curves for MFΔML models from Figure 3 performed above.

The outliers in the plots of Figure 4 warrant some discussion of possible reasons. The large difference in predictions could arise due to a lack of diversity in the training data. Homogeneity in the training data results in the ML models ending up being overfitted to the simplistic training data and struggling to make predictions for out‐of‐sample data. Alternatively, outliers in prediction could be due to the complexity of certain molecules being under‐represented in the training dataset, for example, cyclobuta‐1,3‐diene. Even so, as expressed above, the majority of the predictions are close to the reference values as seen by the peaks being centered around 0 kcal/mol.

**FIGURE 5:**
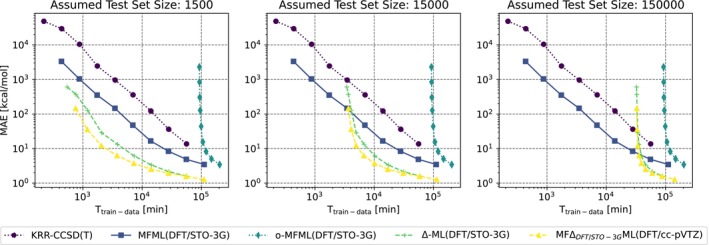
Model MAE versus the time to generate the training data. Three test set sizes are compared.

While these are interesting results about the capabilities of both MFML and MFΔML methods, it becomes pertinent to also account for the time‐cost associated with these different models when predicting DLPNO‐CCSD(T) energies. Figure 5 depicts the model MAE as a function of generating the training data for the collection of ML models that are compared in this work. This comparison is made for the single‐fidelity KRR, MFML, and o‐MFML models built with DFT/STO‐3G baseline fidelity, the Δ‐ML model with the QC‐baseline fidelity, and the MFΔML model with the DFT/cc‐PVTZ fidelity. For the MFML model, the training data cost accounts for the complete multi‐fidelity training structure, similar to what is discussed in [29]. That is, the cost of training data at all the fidelities used in the MFML model. For the o‐MFML model, the time‐cost also includes the cost of generating a validation set over which the optimization procedure is carried out. For Δ‐ML and MFΔML models the cost includes the time to make the QC‐baseline calculations.

Figure 5 compares the time‐cost versus MAE for three hypothetical test set sizes, that is, 1.5k, 15k, and 150k samples. The actual MAE values are calculated over the fixed test set of 1.5k samples. However, since the MAE values reported are for an average of over ten runs, it is expected that the model MAE would be similar for a larger test set. The interesting thing to note is the time‐cost of generating the training data. In cases where one needs to predict energies for a few geometries, 1.5k in this case, the MFΔML model performs the best. As one increases the test set size, the time‐cost of making the QC‐baseline calculations for the Δ‐ML and MFΔML models outweighs the potential benefit of the method. In contrast, the MFML model is unaffected by the size of the test set. This is due to the fact that the MFML approach also predicts the baseline fidelity rather than using QC computed values. In large test set size regimes, this sets the MFML to be the more efficient method. The o‐MFML method, across the different test set sizes, is the most expensive model to build. This is expected since the cost of the validation set is affected by the target fidelity, which in this case is the DLPNO‐CCSD(T), an expensive QC method. Table 1 reports the time‐costs in minutes for the different ML models in contrast to using conventional QC computations for the DLPNO‐CCSD(T) fidelity. The ML models are built with $2^{9}$ training samples at the target fidelity of DLPNO‐CCSD(T). It is evident that the use of any ML method is better than the use of conventional QC computational methods. Notice that the time‐costs for KRR, MFML, and o‐MFML are fixed regardless of the size of the test set. The Δ‐ML and MFΔML, although lower in model MAE are sensitive to the size of the test set. To make this clearer, we also present in the table a test set size of 1.5 million samples. In contrast, the MFML model is unaffected by the size of the test set since even the $f_{b}$ fidelity is predicted with an ML model.

**TABLE 1:** Time‐costs (in minutes) for different sizes of the test set. The reference cost of using DLPNO‐CCSD(T) conventional computation is contrasted alongside. For the ML models, the time‐cost is computed for $N_{\text{train}}^{\mathrm{CCSD}(T)}=2^{9}$ with the remaining multi‐fidelity data structure being accounted for as expressed in the main text. The values in the parenthesis denote the MAE of the ML models. It is to be noted that the Δ‐ML and MFΔML models also have the cost of the QC‐baseline fidelity.

Evaluation size	DLPNO‐CCSD(T)	KRR	Δ‐ML	MFML	o‐MFML	MFΔML
1500	$1.64\times 10^{5}$	$5.61\times 10^{4}$ (13.64)	$5.66\times 10^{4}$ (1.59)	$1.11\times 10^{5}$ (3.46)	$2.04\times 10^{5}$ (3.44)	$1.11\times 10^{5}$ (1.28)
15,000	$1.64\times 10^{6}$		$5.95\times 10^{4}$ (1.59)			$1.14\times 10^{5}$ (1.28)
1,50,000	$1.64\times 10^{7}$		$8.85\times 10^{4}$ (1.59)			$1.43\times 10^{5}$ (1.28)
15,00,000	$1.64\times 10^{8}$		$3.79\times 10^{5}$ (1.59)			$4.33\times 10^{5}$ (1.28)

## 4 Discussion

After the time‐cost assessment of the different ML models, these can be further used to study their predictive capabilities over certain datasets. To this end, the trained MFML model with DFT/STO‐3G baseline fidelity, and the MFΔML models with the DFT/STO‐3G QC‐baseline and DFT/ccpvtz baseline fidelity were evaluated over three specific datasets, that is, atmospheric molecules (Atmos), Conjugated molecules, and Isomers, which were also used in the previous study for validation [21]. It is important to note that some of the configurations in these additional evaluation sets were already present in the training set due to the extension of the previous dataset. We visualized all these duplicate molecules using VMD package [50] and removed identical conformations from the validation sets. However, since the optimization of structures at the DFT level does not always yield the global minimum structure, the same molecule may appear in different conformations in the training and test sets. These conformations have different energies and are still retained for evaluation, especially for the isomer test set. The ML models are trained with $N_{\text{train}}^{\mathrm{CCSD}(T)}=512$ with the remaining multi‐fidelity structure built as explained in Section 2.2.4. The training samples are chosen at random from the training dataset, ensuring that a proper multi‐fidelity structure is retained. The model MAE for this set‐up on the original test set is reported in Table 2 along with its performance on the additional datasets.

**TABLE 2:** MAE in kcal/mol of predictions for the MFML and MFΔML models built with $N_{\text{train}}^{\mathrm{CCSD}(T)}=512$ for the original test set of 1500 samples and for the additional validation datasets. A random selection of training samples was chosen from the training dataset used to generate the learning curves from Figures 2 and 3.

Dataset	MFML	MFΔML
Original test set	3.01	1.28
Atmos	9.60	3.47
Conjugated	8.78	1.69
Isomers	1.48	0.42

**FIGURE 6:**
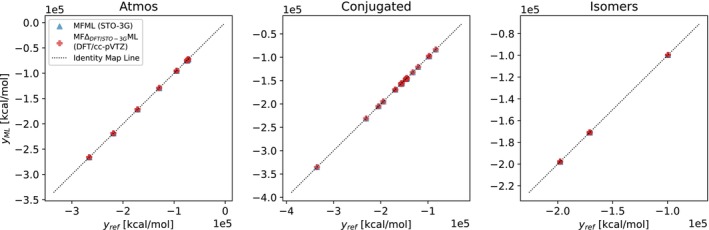
Reference versus ML‐predicted DLPNO‐CCSD(T) energies for the three special test sets for MFML and MLΔML models. Due to the large values of the energies, the axis values are reported in scientific notation. Each axes' tick is to be multiplied with $10^{5}$ for the actual values of the energies, as depicted on the margins.

The predictions of the MFML and MFΔML models for the Atmos, Isomers, and Conjugated datasets are compared to the reference DLPNO‐CCSD(T) values in Figure 6 in the form of a scatter plot with the x axis representing the reference energies while the y axis reports the ML predicted values. For all cases, an identity mapping line, which is the ideal prediction‐reference line, is provided for easy reference. For the three unseen test datasets that the MFML and MFΔML models are tested on, the predictions and reference energies show good agreement, with all the scatter points being on the identity map line. Since the Atmos, Isomers, and Conjugated datasets have very few data points, it is to be estimated from the discussion of Figure 5 that the MFΔML would be more beneficial due to the smaller number of QC‐baseline calculations needed.

**FIGURE 7:**
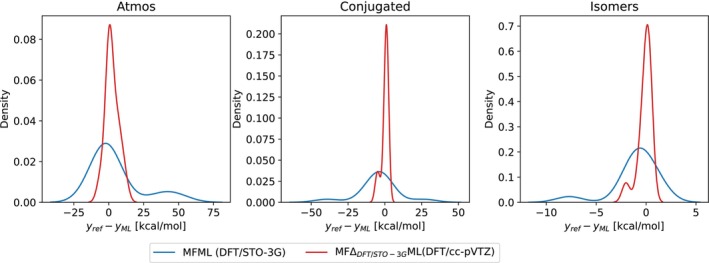
Distribution of the difference between reference and predicted energies for MFML and MFΔML models studied in this work. Note that the different distribution plots have different scaling of the x and y axes to aid better visualization of the distributions.

To better assess this benefit, the distribution of the difference in reference and predicted DLPNO‐CCSD(T) energies are presented in Figure 7 for the MFML and MFΔML models from the above discussion. Consider the case of the Atmos dataset shown in the left‐hand side plot of the figure. The prediction of the MFML model shows a wider plateau skewed towards the negative x‐axis, indicating an over‐estimation of the DLPNO‐CCSD(T) energies. However, with the MFΔML model, the distribution is symmetric around 0 kcal/mol with a distinct peak with most of the deviation from predictions being within ±10 kcal/mol. Similar observations can be made for the Conjugated and Isomers datasets. MFΔML results in narrower distributions of the difference in comparison to the MFML model. This is anticipated since the MFΔML method explicitly contains information about the molecule, albeit at a lower fidelity, in this case, the STO‐3G fidelity. This results in good agreement between the DLPNO‐CCSD(T) reference and the MFΔML model predictions. In order to assess the sensitivity of the composition of the training dataset on the accuracy of the ML models for these additional validations, the models were trained for 5 random training data compositions. Figure S5 in the supplementary document plots the MAEs for each of the additional validation datasets. Acceptable standard deviations of the MAE values are observed and therefore one can safely rule out high sensitivity to the composition of the training dataset.

**FIGURE 8:**
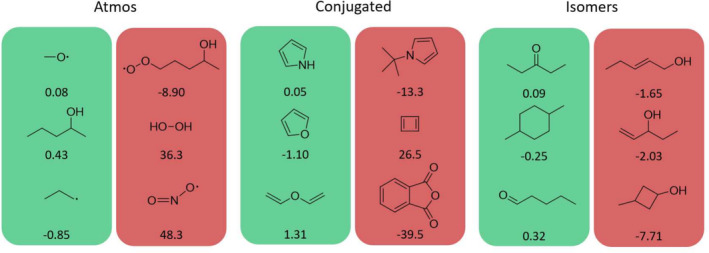
Three best (green background) and three worst (red background) MFML model predictions of each validation set. The differences (true value minus predicted value) are given in the units of kcal/mol.

**FIGURE 9:**
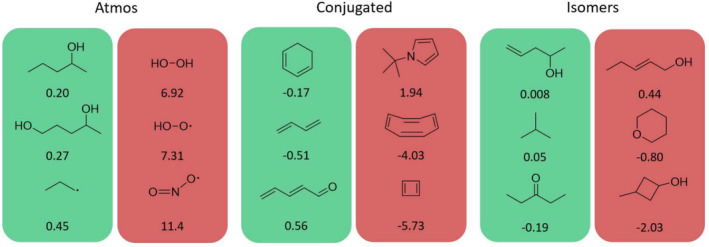
Three best (green background) and three worst (red background) MFΔML model predictions of each validation set. The differences (true value minus predicted value) are given in the units of kcal/mol.

A visual representation of the performance of the multi‐fidelity models can be studied on these additional validation datasets. In particular, one can visualize the best and worst performances of the two multi‐fidelity models in terms of the largest deviation the models predict with respect to the reference DLPNO‐CCSD(T) energies on the Atmos, Conjugated, and Isomers datasets. Figure 8 depicts such a visual for the MFML model. For each of the additional datasets that the model is evaluated on, the three molecules with the lowest MAE and three molecules with the largest MAE are reported in units of kcal/mol. A similar analysis is presented in Figure 9 for the MFΔML model. The two models show a certain consistency here. For the structures that are difficult to predict by one model, the other model usually also gives a large difference between prediction and reference. In general, both models performed best in the isomer set. It should be noted that although this test set contains molecules that are also part of the training set, their conformations are not the same. Moreover, these molecules are actually not always the ones with the best energy compared to the reference energies.

**FIGURE 10:**
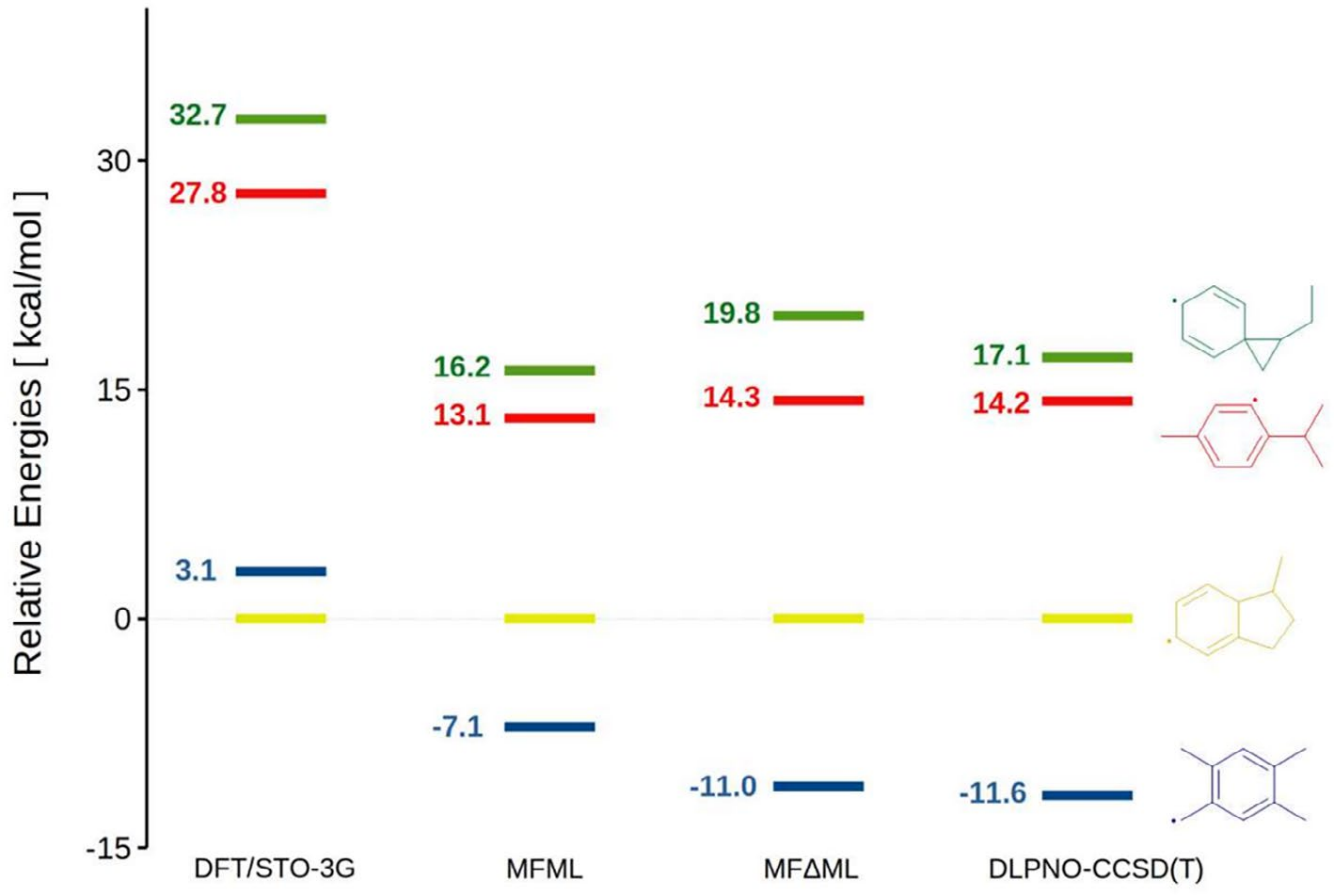
Relative energies of four ${\text{C}}_{10}{\text{H}}_{13}$ isomers. The energy of one of the isomers is selected as the reference (shown in yellow), and the relative energies of the other three isomers obtained using different methods are shown in green, red, and blue, respectively.

In addition to the above comparisons, we picked an example from the original test set to further demonstrate the ability of the present model to distinguish isomeric structures. As shown in Figure 10, compared with the reference ${\text{C}}_{10}{\text{H}}_{13}$ molecule, B3LYP‐D3(BJ)/STO‐3G unexpectedly overestimates the energies of the remaining three isomers, even resulting in an incorrect relative energy order. In particular, the two isomers with the lowest relative energies have an energy difference of 3.1 kcal/mol, while the energy gap at the DLPNO‐CCSD(T) level of theory is 11.6 kcal/mol. However, the STO‐3G basis set serves as the baseline for our ML model and provides general information on molecular energies at a very low cost, although it does not provide precise energies. The present MFML model based on this lowest fidelity, however, is able to correct the relative energy trend. Furthermore, the MFΔML model not only restores the correct relative order, but also obtains results that are numerically close to those of the DLPNO‐CCSD(T) reference. This finding showcases the potential of the present multi‐fidelity models in distinguishing and identifying isomers.

## 5 Conclusions

In this work, different ML approaches starting at the single‐fidelity KRR and including the MFML, o‐MFML, Δ‐ML, and MFΔML schemes were studied in their efficiency to predict DLPNO‐CCSD(T) energies of small organic molecules. The time‐cost of generating the training data and its effect on the overall model accuracy was studied for the different ML models. This study indicates that the MFML method is preferable when a large number of evaluations of the ML model are required. For a smaller number of predictions, the MFΔML method was seen to be more effective. Moreover, the MFML and MFΔML models were evaluated on validation datasets of atmospheric, conjugated, as well as isomeric molecules. In all these cases, the MFΔML method showed good agreement with the reference DLPNO‐CCSD(T) energies, resulting in a positive outlook on the use of the method for further application. Overall, this work provides a strong footing for the use of multi‐fidelity methods in the application of coupled cluster energy predictions of thermochemistry. In addition, this work demonstrates that the use of multi‐fidelity methods increases overall model accuracy. In cases such as predicting energies for a very large dataset, the use of MFML can be more efficient. The results of this work are comparable to previous work by some of us in [20, 21] with the MFML being a cheaper alternative to the Δ‐ML method described therein. Here, we utilized a cheaper and smaller basis set size to further decrease the computational cost associated with the training data for ML models.

A challenge of the existing work is the sensitivity of the ML models used herein to training data. This is a general challenge for ML methods and certainly work is progressing to produce generalized ML‐potentials which can be used for several applications [51, 52, 53]. Another possible limitation of the work presented herein is the sensitivity of the ML models to the geometry optimization procedure carried out to generate the training data itself. Research in the future could attempt to study this relation and provide key insights into the use of fine‐tuned optimization for the ML‐QC pipeline. Further, since the multi‐fidelity hierarchy structure assumed in this work is one‐dimensional, a possible direction that can be pursued is the effect of building fidelity hierarchy with several dimensions, as was demonstrated in [26] wherein both the level of theory and basis set choice were used to construct a multi‐dimensional multi‐fidelity model. A time‐cost assessment of such an approach combined with training set sizes optimization, such as the one in [32, 36] can potentially provide a better understanding of how the multi‐fidelity method works across this form of a fidelity structure and its efficiency thereof. Yet another area of focus can be understanding how different forms of geometry optimization in the pre‐processing stage would affect the overall model accuracy, since the mapping from the coordinates to the property to be learned would change based on how the geometries are produced. Furthermore, a systematic study of the outliers from Figure 4 can be performed to better gauge whether this is due to model artifacts or special chemistry of the molecules themselves. Such a study would also need to assess the molecular descriptors, such as varying several parameters of the SLATM representation.

## Supporting information




**Data S1.** Supporting Information.

## Data Availability

Research data are not shared.
