# Supplementary material for: Predicting Molecular Energies of Small Organic Molecules With Multi‐Fidelity Methods
Source: J Comput Chem. 2025 Mar 4;46(6):e70056. doi: 10.1002/jcc.70056 (PMC11877263; doi:10.1002/jcc.70056)
Supplement: Supplementary file 1 — Data S1. Supporting Information. [file JCC-46-0-s001.pdf]

Supplementary Information:
Predicting Molecular Energies of Small Organic Molecules with Multifidelity Methods

Vivin Vinod[1], Dongyu Lyu[2], Marcel Ruth[3], Ulrich Kleinekathöfer[2], Peter R. Schreiner[3], and Peter Zaspel[1,†]

[1]School of Mathematics and Natural Sciences, University of Wuppertal, 42119 Wuppertal, Germany
[2]School of Science, Constructor University, 28759 Bremen, Germany
[3]Institute of Organic Chemistry, Justus Liebig University, 35392 Giessen, Germany
[†]Correspondence: zaspel@uni-wuppertal.de

January 13, 2025

S1 Data distribution

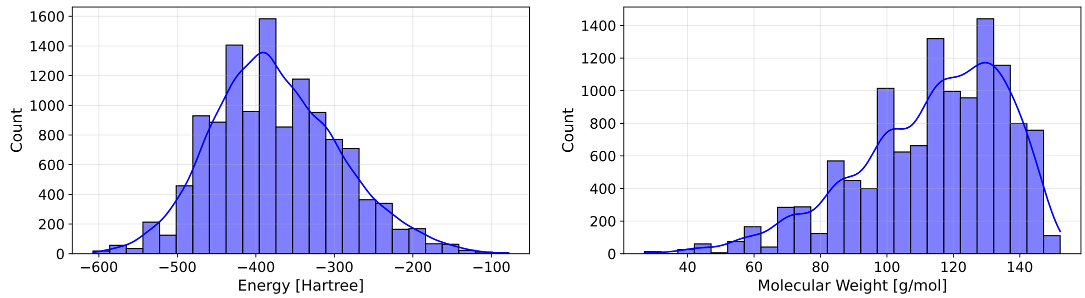

Figure S1: Distributions of the DFT energies and molecular weights.

The molecular energy and weight distributions of the complete dataset we selected, which contains 12,340 molecules or radicals, are shown in Figure S1. Here, we plot the energies computed at the B3LYP-D3(BJ)/cc-pVTZ level of theory, as our dataset includes the energies of all geometries evaluated at this level. The molecular energy follows an approximately normal distribution, indicating the randomness of the molecules selected during the construction of the dataset. The molecular weight exhibits a skewed distribution due to our restriction that the selected molecules

must not exceed 10 heavy atoms, which ensures the high-level quantum chemical calculations can be completed within a relatively reasonable timeframe.

S2 Additional ML Results

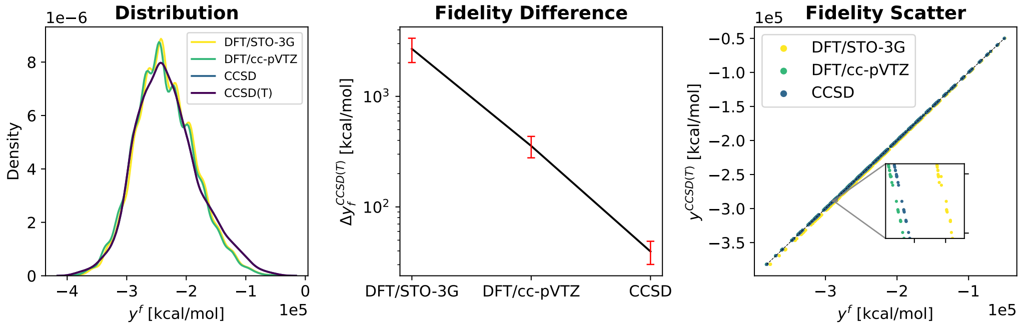

Figure S2: Preliminary multifidelity data analysis of the monomers for the different fidelities used in this work.

A preliminary data analysis of the multifidelity training structure is shown in Figure S2. This is to keep with the recommendation prescribed by some of us in Ref. [2] to check for discrepancy in the multifidelity data structure, which could cause issues with the training of the MFML models. The left-hand side of the figure shows the distribution of the energies for different fidelities. The CCSD and CCSD(T) fidelities are nearly identical in distribution and show a normally distributed landscape of the energies. The cheaper DFT fidelities show several spikes in the data distribution. The middle pane of Figure S2 shows the absolute difference of a given fidelity f w.r.t. the target fidelity CCSD(T). That is, $\Delta_f^{CCSD(T)} = |y^{CCSD(T)} - y^f|$ for varying f. The difference to the target fidelity is seen to be monotonically decreasing with increasing accuracy of the fidelities. The error bars of the plot indicate the standard deviation of the absolute differences. The right most plot of Figure S2 shows the scatter of the energies of a fidelity with respect to the target fidelity energies at CCSD(T) for the training data. Since the energy range covered is quite large, $10^5 - 4 \cdot 10^5$ kcal/mol, the scatter is not clearly visible. To aid this, an inset is provided around $2.8 \cdot 10^5$.

Figure S3 compares the learning curves for Δ-ML with different baselines. The results indicate that the closer the QC-baseline is to the target fidelity, the lower the error can get. Indeed, the case for the CCSD QC-baseline reports the MAE 0.2 kcal/mol with $N_{train}^{CCSD(T)} = 512$, which is similar to the case reported in Ref. [1] for a similar number of training samples where the Δ-ML approach is used to learn the perturbative difference between CCSD and CCSD(T) fidelities.

The time-cost comparisons of the different Δ-ML models is presented in Figure S4 for differing QC-baselines. Three test set sizes are shown: 1,500, 15,000, and 150,000. With increasing proximity of the QC-baseline to the target fidelity, although the MAE decreases, the cost of implementing the Δ-ML model for new predictions becomes unreasonable. With a large test set size, the use of the costlier QC-baselines can not be justified as seen from the plot for 150,000 test set size, with the Δ-ML model with CCSD QC-baseline being two orders of magnitude costlier than the one with DFT-STO3G as the QC-baseline. This high cost is associated with the cost of making

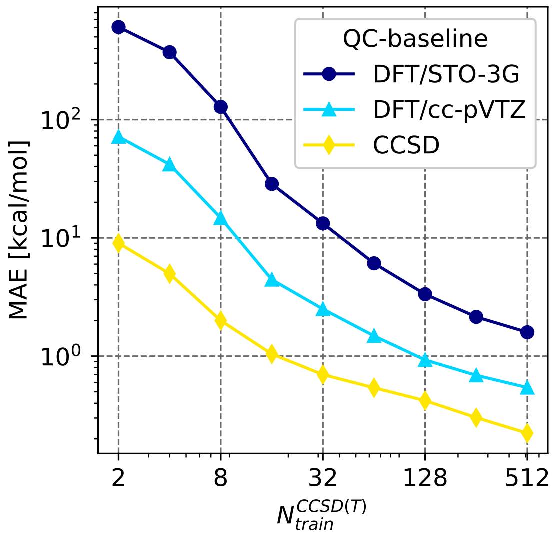

Figure S3: Learning curves for Δ-ML approach of KRR with different QC-baseline for the target fidelity CCSD(T). It is observed that the closer the QC-baseline is to the target fidelity, the lower the model error is.

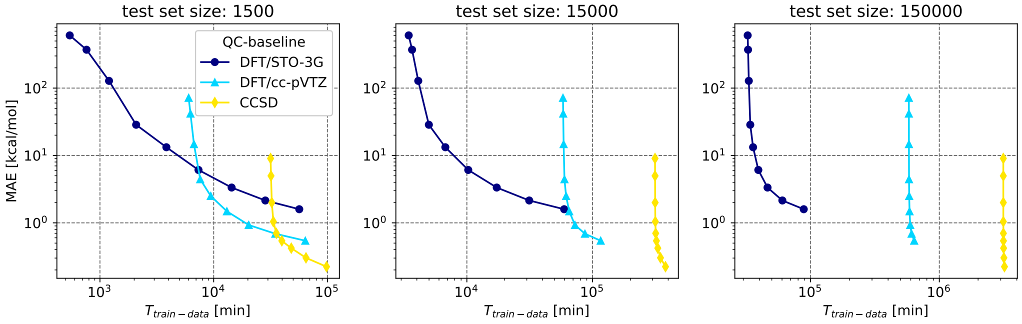

Figure S4: Training data time-cost for Δ-ML approach of KRR with different QC-baseline for the target fidelity CCSD(T). The cost of the QC-baseline calculations is considered for different test set sizes.

the QC-baseline calculations, which are then added to the prediction of the difference between the QC-baseline and target fidelity.

Figure S5 shows the MAE values of the MFML and MFΔML models for the additional validation datasets when different train-test splits are used. The error bars are standard deviations of the MAE centered around the mean MAE achieved for five random choices of training data. The results indicate that the Atmos dataset is highly sensitive to the choice of training data used, more so than the others.

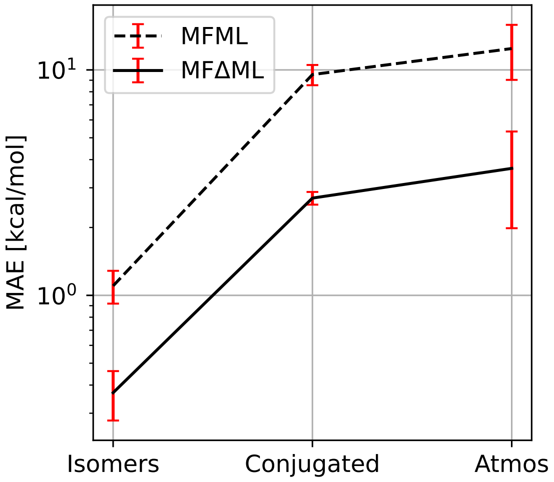

Figure S5: MAE values for the MFML and MFΔML models built with different shuffling of the original training dataset when predicting over the additional validation datasets.

References

[1] Marcel Ruth, Dennis Gerbig, and Peter R. Schreiner. Machine Learning of Coupled Cluster (T)-Energy Corrections via Delta Δ-Learning. *J. Chem. Theory and Comp.*, 18(8):4846–4855, 2022. doi:10.1021/acs.jctc.2c00501.

[2] Vivin Vinod, Sayan Maity, Peter Zaspel, and Ulrich Kleinekathöfer. Multifidelity machine learning for molecular excitation energies. *J. Chem. Theory Comput.*, 19(21):7658–7670, 2023. doi:10.1021/acs.jctc.3c00882.
